# Sleep Disordered Breathing in Pregnancy and Adverse Maternal Outcomes—A True Story?

**DOI:** 10.17925/usrpd.2017.12.02.19

**Published:** 2017-10-30

**Authors:** Margaret H Bublitz, Ghada Bourjeily

**Affiliations:** 1. Department of Medicine, Warren Alpert Medical School of Brown University, Providence, RI, US; 2. Women’s Medicine Collaborative, The Miriam Hospital, Providence, RI, US; 3. Department of Psychiatry and Human Behavior, Warren Alpert Medical School of Brown University, Providence, RI, US

**Keywords:** Pregnancy, sleep disordered breathing, obstructive sleep apnea, maternal outcomes, preeclampsia, maternal morbidity

## Abstract

Pregnancy may predispose women to the development or worsening of sleep disordered breathing. Recent studies have shown a significant association between sleep disordered breathing and adverse pregnancy-related outcomes including gestational diabetes, preeclampsia, and severe maternal morbidity including pulmonary edema, cardiomyopathy, congestive heart failure, and admissions to the intensive care unit. More research is needed on the mechanisms linking sleep disordered breathing to adverse pregnancy outcomes. Large trials that examine the impact of therapy for sleep disordered breathing during pregnancy on pregnancy outcomes are also needed.

Pregnancy is associated with dynamic physiologic changes that may predispose women to the development, or worsening, of sleep disordered breathing. Such changes include upper airway narrowing and edema that are related to decreased oncotic pressure, the presence of estrogen receptors in the upper airway, and reduced functional residual capacity and chest wall compliance.^1^ In addition, the obesity epidemic in many countries in the developed world, including the United States, affects women of childbearing age,^2^ and is likely a major contributor to the presence of obstructive sleep apnea (OSA) in this young population. Based on these facts, and with data from the Wisconsin Sleep Cohort, suggesting a high prevalence of OSA in pre-menopausal women,^3^ it is likely that a subset of pregnant women are entering pregnancy with OSA, but this diagnosis probably remains under-recognized.^4^

Recent studies have shown a significant association between OSA and adverse pregnancy-related outcomes. Associations between OSA and gestational hypertensive disorders have been shown in prospective studies of pregnant women with obesity,^5^ as well as in the general pregnant population.^6^ A similar association was found between sleep disordered breathing and gestational diabetes in prospective and cross-sectional cohorts.^6,7^ In addition, a high prevalence of sleep disordered breathing was demonstrated in cohorts of pregnant women with a diagnosis of gestational diabetes.^8,9^ In addition, national registry and population-based samples around the world^10–12^ have shown a significant association of sleep disordered breathing with gestational hypertensive disorders and gestational diabetes.

A recent study by the authors has shown that the impact of having a diagnosis of OSA extends beyond pregnancy-specific complications, and is linked to severe maternal complications.^13^ Data from the National Perinatal Information Center dataset, collected from 95 perinatal centers across all geographic areas in the United States from 2010–2014, were examined for an association between a diagnosis of OSA and severe maternal morbidity based on the International Classification of Diseases collected at the time of delivery. The sample consisted of nearly 1.6 million pregnant women with an overall rate of OSA of 0.12%. The study showed a significant association between an OSA diagnosis and pulmonary edema, cardiomyopathy, congestive heart failure, and hysterectomy. In addition, women with OSA were more likely to be admitted to the intensive care unit and to require a longer hospital stay. All of these associations were significant after adjusting for an extensive list of covariates, which included obesity, pre-gestational diabetes and pre-gestational hypertension, substance use, and multiple comorbidities.^13^

The literature to date seems to be consistent in showing significant associations of sleep disordered breathing with serious complications during pregnancy, independently of body mass index and other potential risk factors. Though pregnancy is short-lived and self-limited and many pregnancy-related physiologic changes are reversible, conditions that occur during pregnancy may impact the long-term health of both the mother and her offspring. Data from the past decade suggest that conditions such as preeclampsia and gestational diabetes may impact maternal risk for cardiovascular^14^ and metabolic outcomes later in life,^15^ and increase the risk of neonatal complications such as prematurity and growth restriction. The latter conditions may in turn impact the offspring’s long-term health, and lead to a significant financial and societal burden. Hence, it is the association between sleep disordered breathing and adverse outcomes that make the recognition of this condition in pregnancy a priority. It is important to keep In mind, however, that though experimental laboratory data^16,17^ of interventions with positive airway pressure (PAP) therapy, as well as case series,^18,19^ show significant overnight improvement in blood pressure, cardiac output, and symptoms of sleep disordered breathing, data showing similar improvement in larger randomized controlled trials are lacking. Future research needs to focus on mechanistic pathways linking the association of sleep disordered breathing with adverse maternal outcomes to help identify potential targets for intervention. In addition, large trials that examine the impact of therapy for sleep disordered breathing during pregnancy on pregnancy outcomes are also needed.
